# Advancing State and Local Home Modification Practice and Policy: Findings From Aging Network Surveys

**DOI:** 10.1093/geroni/igab046.1885

**Published:** 2021-12-17

**Authors:** Julie Overton, Jon Pynoos, Emily Nabors, Damon Terzaghi, Elizabeth Blair, Traci Wilson, Bernard Steinman, Suzanne Kunkel

**Affiliations:** 1 University of Southern California, Los Angeles, California, United States; 2 University of Southern California, California, United States; 3 USC Leonard Davis School of Gerontology, Los Angeles, California, United States; 4 ADvancing States, Arlington, Virginia, United States; 5 n 4.a, Washington, District of Columbia, United States; 6 USAging, Washington, District of Columbia, United States; 7 University of Wyoming, University of Wyoming, Laramie, Wyoming, United States; 8 Miami University Scripps Gerontology Center, Oxford, Ohio, United States

## Abstract

Home modification (HM) can promote older adults’ functioning as their needs change, reduce fall risks, and support caregivers. A supportive home environment is increasingly important as homes become healthcare delivery sites for home and community-based services (HCBS). HM is funded and administered by disparate agencies, often hindering access to HM services for at-risk older adults who need them the most. The Aging Network (State Units on Aging (SUAs), Area Agencies on Aging (AAAs), and Title VI organizations serving Native American older adults) plays an important but not well understood role in HM. To address this lack of research, the USC Leonard Davis School of Gerontology, ADvancing States, and the National Association of Area Agencies on Aging in cooperation with Scripps Gerontology Center conducted three national surveys, with support from the Administration for Community Living: 1) directors of the 56 SUAs with an 89% response rate; 2) directors of the 618 AAAs with a 79% response rate; and 3) directors of 276 Title VI programs with an 84% response rate. Exemplary practices included HM advocacy through interagency coalitions; state and local plan priority setting; creative HM financing with housing, disability, and health care sectors, including partnerships with Medicaid agencies; and integration of HMs into state and local HCBS, including nursing home transition and caregiver support programs. Findings on the types of HM activities, service delivery barriers, funding sources, collaborations, and targeted populations will inform HM policy and practice for the Aging Network’s critical state and local agencies serving low-income older adults.

